# Acceptability of HPV vaccines and associations with perceptions related to HPV and HPV vaccines among male baccalaureate students in Hong Kong

**DOI:** 10.1371/journal.pone.0198615

**Published:** 2018-06-18

**Authors:** Teris Cheung, Joseph T. F. Lau, Johnson Z. Wang, P. K. H. Mo, Y. S. Ho

**Affiliations:** 1 School of Nursing, Hong Kong Polytechnic University, Hong Kong, China; 2 The Jockey Club School of Public Health and Primary Care, Faculty of Medicine, The Chinese University of Hong Kong, Hong Kong, China; 3 School of Public Health, Zhejiang University, Zhejiang Province, China; Public Health England, UNITED KINGDOM

## Abstract

**Objectives:**

The highly infectious human papillomavirus (HPV) causes both genital warts and cervical cancer in women. In 2009, the prevalence of genital warts in Hong Kong was 203.7 per 100,000 person-years. Cervical cancer, more seriously, was the eight most common cancer among women and girls in Hong Kong, accounting for 2.3% of all new cancer cases in females in 2014. Cervical cancer is a significant global public health problem and HPV is a major risk factor leading to the development of cervical cancer. HPV is also the most common sexually transmitted disease among university students. This is the first study to examine the acceptability of HPV vaccines and associations with perceptions related to HPV and HPV vaccines among the male baccalaureate student population locally.

**Methods:**

A self-administrative cross-sectional survey was used to assess whether male baccalaureate students from eight local Hong Kong universities intended to be immunized for HPV. The study also asked questions concerning how its subjects perceived HPV and HPV vaccines using the Health Belief Model. Data collection spanned from June to September 2015. A multiple stepwise regression model was used to examine associations between cognitive factors and subjects’ intention to take up the HPV vaccine.

**Results:**

A total of 1,004 (83.7%) students aged 18 and 26 participated in this study. 23.3% found vaccinating for HPV acceptable, a level correlating with a number of indicators. Subjects were more likely to find vaccinating acceptable if 1) they knew something about HPV vaccines; 2) they understood that men were susceptible to infection by HPV; 3) they realised they could benefit by HPV vaccination, and 4) they were aware of the arguments for and against HPV vaccination, as disseminated by either the media or peers.

**Conclusions:**

HPV remains a significant public health concern in Hong Kong and China more broadly. This study’s findings show a disconnect between the perceived and actual risk of being infected with the HPV vaccine among male baccalaureate students. This disconnect may be bridged by informing young men of the benefits of their being vaccinated against HPV, by removing the psychological and financial barriers that prevent them from taking up the vaccine and by improving how primary healthcare providers motivate them to get immunized.

## Introduction

Human papillomavirus (HPV) infections are the world’s most common sexually transmitted infections (STI) among men and women in the world [1]. Human papillomavirus (HPV), a highly transmissible virus, is acquired from infected persons mainly through sexual, particularly vaginal and anal, intercourse [2]. A recent systematic review (2001 and 2012; of 37 studies: 15 from Europe; 10 from North America; 1 from Canada; 5 from Asia; 3 from South America; 2 from Australia and 1 multiregional) of the prevalence of genital warts reported an overall (male and female combined) annual incidence of anogenital warts (AGWS) ranging from 160 to 289 per 100,000 [3]. In the United States, approximately 3 to 4 million cases of genital warts occurred in men each year, at a peak rate of 500 per 100,000 in age between 25 and 29 [4]. In 2009, the incidence of genital warts for males in Hong Kong was estimated to be 292.2 per 100,000 persons per year [5]. Men more prevalently report AGWs than women [3, 5]. According to these prevalence estimates, Hong Kong ranks third among all countries in the systematic review conducted by [3] and is the *most* at-risk city for genital warts. Men are also at risk for other HPV- related cancers including penile, anal and oral cancers [6]. Penile cancers contributed to a very small fraction (0.4% to 0.6%) of all male cancers in the United States and Europe. Nevertheless, the incidence of penile cancers is almost 20-fold among Asia men [6]. Penal and oral cancers are also known to be more prevalent among men who are having sex with men (MSM) [7] than among general male population [8]. Sexual behaviours including multiple sex partnership and male-homosexual oral sex are associated with increased risks of HPV-associated cancers. Recent evidence has estimated that 60% to 70% of oropharyngeal squamous cell cancer (OPSCCs) was associated with HPV infection in the United States [9]. The Centers for Disease Control and Prevention also estimated that over 15,000 HPV-associated OPSCCs were diagnosed annually in the United States and the oropharynx (head and neck) cancer have increased in incidence [10]. The number of lifetime sexual partners in heterosexual men is also a strong risk factor of HPV[11], regardless of their age[12].

A systematic review conducted in Europe [13] between 1990 and 2010 concluded that those between 13 and 20 years old teenage boys and girls had low awareness of HPV infections (5.4% to 6.6%) and poor knowledge towards HPV vaccines (5.8% to 1.1%). Makwe et al [14] echoed the findings emerging from this systematic review by reporting that less than 18% of the undergraduate female students in a Nigeria’s study (n = 368) had ever heard of HPV infections and HPV vaccine. Less than 11% of them were aware that HPV infections may cause cervical cancer.

Cervical cancer is the eight commonest cancer among females in Hong Kong, being responsible for 2.3% of all new cancer cases in women and girls in 2014. In 2014, 472 new cases of cervical cancer were diagnosed. The crude incidence rate was 12.1 per 100,000 of the female population. In 2015, 169 women died from cervical cancer, representing 2.8% of female cancer deaths [15].

Although HPV vaccine is widely available for male and female in Hong Kong, only a paucity of research has examined gender differences in acceptability of HPV vaccine among baccalaureate students in Asia, China and Hong Kong. Recent research reported that more female university students were taking proactive steps towards HPV vaccination than male counterparts and there were significant gender differences in HPV-related beliefs and attitudes among university students [16]. A recent cross-sectional study conducted in Hong Kong revealed that approximately 88% of undergraduates in both sexes had heard about cervical cancer. 92.2% heard about HPV vaccine, nonetheless; they had poor knowledge of HPV vaccination in general [17].

### HPV vaccines

The human papillomavirus (HPV) vaccine (also called “cervical cancer vaccine”) is a prophylactic vaccine designed to prevent cervical cancer and other HPV-related cancers or diseases.

Currently, three HPV vaccines, namely, Cervarix (a 2-valent vaccine against HPV-16 & 18), Gardasil-4 (a 4-valent vaccine against HPV-6, 11, 16 &18) and Gardasil-9 (a 9-valent vaccine against HPV-6, 11, 16, 18, 31, 33, 45, 52 & 58) have been registered in Hong Kong for the prevention of cervical cancer and/or other HPV-related diseases [15]. All three vaccines are capable of preventing infections from HPV-16 and 18, which account for about 70% of all cervical cancers worldwide. The 9-valent vaccine covers five additional HR-HPV, viz. HPV-31, 33, 45, 52 & 58, which are associated with around 90% of cervical cancer in Hong Kong and worldwide. The 4- valent and 9-valent vaccines also protect against two LR-HPVs, HPV-6 and 11, which are associated with anogenital warts [18].

HPV vaccination is recommended routinely for boys and girls aged between 11 and 12 years old [19]. It can also be administered at a younger age before recipients start exploring sex [20]. HPV vaccine gives the best protection to the most ‘at risk’ groups during the adolescence period before exposure of HPV infections. Currently, the Hong Kong Family Planning Association of Hong Kong has launched a free cervical cancer vaccination pilot scheme targeting at girls aged 9 to 18 for a period of three years from 2016 to 2019. Approximately 50% of all new HPV infections occur among young people between the ages of 15 and 24 [21]. University-aged students represent an important catch-up population [16, 22]. Therefore, secondary school students pupils and the university student population are most at risk [23]. Nevertheless, little research looks into the acceptability of the HPV vaccine among male student populations in Hong Kong and elsewhere in Asia. How do these young men see HPV vaccine?

This is, then, the aims of this study to examine the acceptability of HPV vaccines and associations with perceptions related to HPV and HPV vaccines among male baccalaureate students’ in Hong Kong.

### Research hypotheses

1. Perceived susceptibility of contracting HPV will increase the likelihood of taking up HPV vaccination;
2. Perceived severity of HPV is positively associated with the intention to take up HPV vaccination;
3. Knowledge of HPV vaccines is associated with acceptability towards HPV vaccination;
4. Perceived self-efficacy is associated with the intention to take up HPV vaccination.

## Materials and methods

### Sample and data collection

The study implemented a cross-sectional self-administrative survey. Convenience sampling recruited study participants from eight local universities in Hong Kong. To be included subjects needed to be male, currently a full-time student enrolled in a local university, aged between 16 and 29 and able to read Chinese, the language of the survey. Since all participants were 16 years old or above, no parental / guardian consent were required. Part-time students and non-readers of Chinese were excluded from the study. Flyers were posted up on notice boards in canteens and communal areas on-campus one week before commencement of data collection. Mass email invitations were delivered to subject lecturers in all research sites to seek permission to allow research assistants and student helpers to promote this study before and after classes. Participants were recruited within university campus by research assistants and student helpers who introduced the aims and objectives of this study. Confidentiality were assured. Participants were given a self-addressed blank envelope with an information sheet and our survey. Participants were asked to return the completed survey in a sealed envelope to the research assistants / student helpers on-site. Returning the completed survey inferred implied consent. This study was approved by the Human Subjects Ethics Sub-committee, the Institutional Review Board of a local university in Hong Kong.

A total of 1,200 eligible baccalaureate students were recruited from eight local universities to participate in this study from June to September 2015. 191 students refused to participate and 5 students missed out data in their survey returns and were removed from analysis.

### Health Belief Model and outcome measure

Extensive research has been conducted using HBM to examine the attitudes and beliefs about HPV and HPV vaccination. Nonetheless, there seems to be a paucity of research examining the acceptability of HPV vaccination among university male students in the globe [24–26]. This study incorporated the Health Belief Model (HBM) to explain and predict health behavior. The HBM is a theoretical framework commonly used to guide public health interventions [27]. HBM explicates that individuals who feel susceptible to serious consequences of a health issue may change their behavior when the benefits outweigh the barriers or costs of adopting a new behavior [28]. Perceived susceptibility, perceived severity, perceived benefits, perceived barriers, cues to action, and self-efficacy were the constructs within the HBM.

The survey filled in by participants had 21 items and closely followed the format of Lau’s comparable study [29]. It solicited socio-demographic information including any personal history of sexually transmitted infection (STI) (both symptoms and diagnoses) and participants’ number of sexual partners over the past six months. Participants expressed views on the acceptability of vaccination 5-point Likert scale from 0–5 (0: highly acceptable, or “very likely” they would take up vaccination to 5: highly unacceptable, or “very unlikely” they would take up vaccination). Four questions were asked if participants suffered HPV-related symptoms in the past year including 1) Do you experience any burning sensation in voiding or micturition? (yes/no); 2) Do you notice any presence of white / yellowish urethral secretion? (yes/no)? 3) Is there any prominent growth around the genital skin or mucosa? (yes/no); 4) Is there any prominent lumps around the genital skin or mucosa? (yes/no). The survey also asked respondents what they knew about and how they thought about HPV and HPV vaccines (or how they “cognized” these in terms of the HBM). Six composite indicator variables were constructed by counting the number of affirmative item responses reflecting how far respondents believed they were susceptible to infection by HPV (0 to 3), how severe they believed the infection was (0 to 3), whether they perceived vaccination could benefit them (0 to 5), how hard they thought it would be for procure vaccination (0 to 5), whether they thought they could vaccinate themselves easily (0 to 2) and whether they were cued to take the HPV vaccination by the media, doctors and peers (0 to 2).

### Statistical analysis

Univariate analysis first determined odds ratios (ORu) for the likelihood of subjects getting vaccinated. Background variables that could be candidates for significantly affecting this likelihood for subjects were fit into a multiple stepwise regression model to generate a multivariate odds ratio (ORm). Associations between the subjects’ cognitive factors and the dependent variable (their intention to get vaccinated) were assessed, adjusting for those background variables that were found significant in the multivariate analysis. This derived adjusted odds ratios (AOR). Odds ratios were given at a 95% confidence interval (CI). The statistical analysis was performed using SAS 9.2, with p values of < .05 taken as statistically significant.

## Results

### Background characteristics

A total of 1,004 baccalaureate students participated in this study. The response rate was 83.7%, of which 80.3% came from non-health related faculties. The vast majority of the participants (97.4%) were aged between 18 and 26. The mean age was 22 (SD: 2.30). 32.9% were sexually active, with past year the participants had previous exposure to HPV-related services (e.g. leaflets/pamphlets, educational talks etc), with 6.2% (n = 62) exhibiting self-reported STD-related symptoms and 1.3% (n = 13) actually diagnosed with a sexually transmitted infection (STI) in the past year (Table 1).

**Table 1:** Frequency distributions of the socio-demographic background variables (n = 1004).

	n	%
Background characteristics		
Age group		
16–17	9	0.9
18–26	979	97.5
27–29	16	1.6
Study subject of the participant (Programme enrolled)		
Health related	198	19.7
Non health related	806	80.3
Previous exposure to HPV-related services		
Exposed to HPV-related services before	500	49.8
Never expose to HPV-related services before	504	50.2
STD history		
Self-reported STD-related symptoms in the past year	62	6.2
STD Diagnosed in the past year	13	1.3
Number of sexual partner in the past six months		
0	674	67.1
1	297	29.6
>1	33	3.3

#### Intention to take up HPV vaccines in the next six month (Acceptability)

A total of 23.3% of respondents said they intended to get vaccinated for HPV in the next six months.

#### Perceived severity and perceived susceptibility of HPV infection

The prevalence rate of responding knowing something about HPV on one of the test’s knowledge items ranged from 20.5% to 60.4%. (Table 2). Survey respondents had some common misconceptions, for instance that HPV could be controlled by using antibiotics (66.2%), that HPV had been recently discovered (50.6%), that it was hereditary (74.7%), that it was unlikely to be totally curable (64.5%), and that it was responsible for a mortality rate of over 5%, and that it did not cause anal and penile cancers (51.9% to 63.8%) (Table 2).

**Table 2:** Frequency distributions of variables related to perceived severity and perceived susceptibility of HPV infection (n = 1004).

	n	%
**Knowledge on HPV**		
Heard of HPV before		
Yes	665	66.2
Never heard of HPV	339	33.8
Whether males could be affected by HPV		
Only male	37	3.7
Only female	202	20.1
Both male and female*	606	60.4
Don’t know	159	15.8
Route of HPV transmission		
Both sexual and Mother-to-infant transmission*	379	37.7
Other route/don’t know	625	62.3
HPV was newly found in the last few years		
Yes/don’t know	508	50.6
No*	496	49.4
HPV could be controlled by antibiotics		
Yes/don’t know	665	66.2
No*	339	33.8
HPV is unlikely to be totally cured		
Yes*	356	35.5
No/don’t know	648	64.5
HPV is hereditary		
Yes/don’t know	750	74.7
No*	254	25.3
High mortality rate (>5%)		
Yes/don’t know	798	79.5
No*	206	20.5
Whether genital warts could be caused by HPV		
Yes*	483	48.1
No/don’t know	521	51.9
Whether anal cancers could be caused by HPV		
Yes*	363	36.2
No/don’t know	641	63.8
Whether penile cancers could be caused by HPV		
Yes*	373	37.2
No/don’t know	631	62.8
Number of appropriate response to the above questions on knowledge related to HPV		
0	68	6.8
1	148	14.7
2	98	9.8
3	98	9.8
4	110	11.0
≥5	482	48.0
**Perceived severity of HPV infection**		
Perceived chance of contracting HPV in the future		
Low/very low	683	68.0
Moderate	241	24.0
Very high/high	80	8.0
Perceived damages of HPV infection on physical health		
Low/very low	164	16.3
Moderate	302	30.1
Very high/high	538	53.6
Perceived infectivity of HPV		
Low/very low	208	20.7
Moderate	439	43.7
Very high/high	357	35.6
Number of responses to the above 3 questions reflecting perceived severity^φ^		
0	406	40.4
1	276	27.5
≥2	322	32.1
**Perceived susceptibility of HPV infection**		
Perceived knowledge on HPV		
Low/very low	564	56.1
Moderate	315	31.4
Very high/high	125	12.5
Perceived prevalence of HPV infection among male in Hong Kong		
Low/very low	385	38.3
Moderate	439	43.8
Very high/high	180	17.9
Perceived prevalence of HPV infection among female in Hong Kong		
Low/very low	95	9.4
Moderate	492	49.0
Very high/high	417	41.6
Number of responses to above 3 questions reflecting perceived susceptibility^φ^		
0	502	50.0
1	332	33.1
≥2	170	16.9

*Appropriate response

^φ^Number of affirmative responses (very high/high)

Regarding perceived susceptibility, 41.6% of the participants perceived that the prevalence of HPV infection among women in Hong Kong as high or very high. In contrast, only 17.9% regarded prevalence among males as at this level. 35.6% believed the infectivity of HPV as high or very high. Nevertheless, only 8.0% of the participants perceived a high or very high chance that they could contract HPV in the future. Item responses reflecting the perceived severity of HPV showed that 53.6% of granted the virus was injurious to physical health. 8% thought that infection could cause genital warts and 35.6% penile or anal cancer (Table 2).

#### Perceptions related to HPV vaccines and intention to take up HPV vaccines

Subjects know little of the availability or price of HPV vaccine. Nor did they know that it was delivered in three shots, or the preferred age range for vaccination. The prevalence rate for correct responses to these question items ranged from 12.2% to 39.5% (Table 3). 44.5% thought that the vaccine could prevent genital warts, 42.5% penile and anal cancers and 36.3% another STD than genital warts. 24.1% believed could treat genital warts and 21.3% penile and anal cancers. Regarding perceived barriers to getting vaccinated, the prevalence of agreement with statements came in as shown: ‘‘HPV vaccines are expensive” (57.7%); ‘‘there are side effects” (28.5%); ‘‘it is embarrassing to take up HPV vaccines” (36.0%); ‘‘it is a hassle [or a bother] to take up HPV vaccines” (36.0%), "private doctors do not provide HPV vaccines" (10.7%), "public hospitals do not provide HPV vaccination" (23.0%), ‘‘getting vaccinated for HPV means you are promiscuous” (19.7%) and "men usually won’t agree to get a HPV vaccination" (57.9%). Students thought they were self-efficacious or capable of getting vaccinated for HPV (76.7% or 82.0%, depending on item-wording). Some sources of information were seen as more effective cues prompting vaccination (with the media 35.0%, and doctors’ advice at 23.4% and peers at 9.2%) (Table 3).

**Table 3:** Perceptions related to HPV vaccines and intention to take up HPV vaccines.

	n	%
**Knowledge on HPV vaccines**		
Availability of effective HPV vaccines to men		
No/Don’t know	607	60.5
Yes*	397	39.5
Perceived price per shot (HK$: 1US$ = 7.8HK$)		
<800/>1500/Don’t know	733	73.0
800–1500*	271	27.0
Number of shots required		
1-2/ Don’t know	677	67.4
3*	327	32.6
Duration of protection		
1 year/2-5 years/10 years or above /Lifelong/Don’t know	882	87.8
5–10 years*	122	12.2
Age group suggested for HPV vaccination		
Below 9 years old/Above 27 years old/All age group/Don’t know	658	65.5
9–26 years old*	346	34.5
Number of appropriate response to the above questions on knowledge related to HPV		
0	408	40.6
1	199	19.8
2	132	13.1
3	102	10.2
4	121	12.1
5	42	4.2
**Perceived benefits of HPV Vaccines for preventing and treating diseases related to HPV**		
Perceived efficacy in preventing genital warts		
Not very effective/not effective/Don’t know	557	55.5
Very effective/effective	447	44.5
Perceived efficacy in preventing HPV-induced cancers (penile and anal cancers)		
Not very effective/not effective/Don’t know	577	57.5
Very effective/effective	427	42.5
Perceived efficacy in preventing STD other than genital warts		
Not very effective/not effective/Don’t know	640	63.7
Very effective/effective	364	36.3
Perceived efficacy in treating genital warts		
Not very effective/not effective/Don’t know	762	75.9
Very effective/effective	242	24.1
Perceived efficacy in treating HPV-induced cancer (penile and anal cancers)		
Not very effective/not effective/Don’t know	790	78.7
Very effective/effective	214	21.3
Number of item responses to the above five questions reflecting Perceived benefits of HPV vaccines^φ^		
0	459	45.7
1	75	7.5
2	120	12.0
3	170	16.9
4	31	3.1
5	149	14.8
**Perceived barriers to take up HPV vaccines**		
HPV vaccination is expensive		
Totally disagree/disagree	122	12.2
Totally agree/agree	580	57.7
Don’t know	302	30.1
HPV vaccines could have sides effects		
Totally disagree/disagree	343	34.2
Totally agree/agree	286	28.5
Don’t know	375	37.4
It is embarrassing to take up HPV vaccines		
Totally disagree/disagree	476	47.5
Totally agree/agree	361	36.0
Don’t know	166	16.5
It is troublesome to take up HPV vaccines		
Totally disagree/disagree	425	42.4
Totally agree/agree	361	36.0
Don’t know	218	21.7
Private doctors do not provide HPV vaccination		
Totally disagree/disagree	580	57.8
Totally agree/agree	107	10.7
Don’t know	317	31.6
Public hospitals do not provide HPV vaccination		
Totally disagree/disagree	436	43.5
Totally agree/agree	231	23.0
Don’t know	337	33.6
Taking up HPV vaccine may be seen as a sign of promiscuity		
Totally disagree/disagree	641	63.8
Totally agree/agree	198	19.7
Don’t know	165	16.4
Male usually won’t agree to have HPV vaccination		
Totally disagree/disagree	214	21.3
Totally agree/agree	581	57.9
Don’t know	209	20.8
Number of item responses to the above eight questions reflecting Perceived barriers related to HPV^φ^		
0	196	19.5
1	141	14.0
2	157	15.6
≧3	847	84.4
**Perceived self-efficacy to take up HPV vaccines**		
I am confident that I could take up HPV vaccines if I want to		
Disagree/Don’t know	234	23.3
Agree	770	76.7
I have full control on whether taking up HPV vaccines		
Disagree/Don’t know	181	18.0
Agree	823	82.0
Number of item responses to the above 2 questions reflecting Perceived self-efficacy^φ^		
0	148	14.7
1	119	11.9
2	737	73.4
**Cue to action**		
I have watched media reports promoting HPV vaccines among men		
No/unknown	653	65.0
Yes	351	35.0
Doctor recommended me to take up HPV vaccines		
No/ unknown	769	76.6
Yes	235	23.4
Peer recommended me to take up HPV vaccines		
No/ unknown	912	90.8
Yes	92	9.2
Number of item responses to the above three questions reflecting cue to action received^φ^		
0	606	60.4
1	153	15.2
≧2	245	24.4
**Behavioral intention to take up HPV vaccines**		
Intention to take up HPV vaccines		
Must	57	5.7
High probability	177	17.6
Low probability	608	60.6
Must not	162	16.1

*Appropriate response.

^φ^ Number of affirmative responses (very effective/effective, totally agree/agree, yes)

### Factors associated with acceptability of HPV vaccines

Four background variables were statistically significant for whether respondents intended to get vaccinated: previous exposure to HPV services (ORm = 4.200, 95% CI = 2.985–5.908), the presence of self-reported-STD related symptoms in the last year (ORm = 2.230, 95% CI = 1.257–3.955), an STD diagnosis in the past last year (ORm = 7.531, 95% CI = 2.271–24.972) and respondents’ number of sexual partners (ORm = 1.371, 95% CI = 1.045–1.800). Adjusting for these four variables, the results showed that six knowledge variables were subsequently statistically significant: responses to the items ‘‘HPV is a virus that has been newly discovered in the past few years” (AOR = 2.659, 95% CI = 1.894–3.732), “HPV causes a mortality rate of over 5%” (AOR = 6.806, 95% CI = 4.745–9.763), “HPV may cause anal cancers” (AOR = 5.472, 95% CI = 3.887–7.701), “effective HPV vaccines are readily available to me” (AOR = 6.462, 95% CI = 4.447–9.390), an item capturing the perceived price of HPV vaccine per shot (AOR = 4.389, 95% CI = 3.136–6.142) and an item related to the number of shots required to secure immunity (AOR = 6.881, 95% CI = 4.801–9.861) (Table 4).

**Table 4:** Associations between factors related to HPV/HPV vaccine and the intention to take up HPV vaccines (n = 1004).

	Row %	ORU (95%CI)	AOR (95%CI)
**Knowledge on HPV**			
HPV was newly found in the last few years			
Yes/don’t know	13.8	1	1
No	33.1	3.091 (2.258, 4.231)***	2.659 (1.894, 3.732)***
High mortality rate (>5%)			
Yes/don’t know	14.8	1	1
No	56.3	7.427 (5.299, 10.410)***	6.806 (4.745, 9.763)***
Whether anal cancers could be caused by HPV			
No/don’t know	10.8	1	1
Yes	45.5	6.907 (4.995, 9.552)***	5.472 (3.887, 7.701)***
**Knowledge on HPV vaccine**			
Availability of effective HPV vaccines to me			
No/don’t know	9.1	1	1
Yes	45.1	8.241 (5.863, 11.583)***	6.462 (4.447, 9.390)***
Perceived price per shot (HK$:1US$ = 7.8HK$)			
<800/>1500/don’t know	14.1	1	1
800–1500	48.3	5.723 (4.171, 7.854)***	4.389 (3.136, 6.142)***
Number of shots required			
1-2/don’t know	11.5	1	1
3	47.7	7.005 (5.084, 9.653)***	6.881 (4.801, 9.861)***
**Perceived severity of HPV infection**			
Perceived chance of contracting HPV in the future			
Low/very low	11.7	1	1
Moderate	46.1	6.436 (4.561, 9.081)***	4.949 (3.455, 7.089)***
Very high/high	53.8	8.759 (5.326, 14.406)***	6.492 (3.807, 11.069)***
Perceived damages of HPV infection on physical health			
Low/very low	6.1	1	1
Moderate	27.2	5.740 (2.885, 11.421)***	3.635 (1.787, 7.395)***
Very high/high	26.4	5.522 (2.832, 10.767)***	4.607 (2.329, 9.114)**
Perceived infectivity of HPV			
Low/very low/moderate	31.7	1	1
Very high/high	35.0	2.659 (1.971, 3.588)***	2.729 (1.979, 3.764)***
**Perceived susceptibility of HPV infection**			
Perceived knowledge on HPV			
Low/very low	9.8	1	1
Moderate	31.1	4.179 (2.897, 6.029)***	3.531 (2.357, 5.290)***
Very high/high	64.8	17.037 (10.749, 27.003)***	13.316 (8.049, 22.029)***
Perceived prevalence of HPV infection among male in Hong Kong			
Low/very low	9.1	1	1
Moderate	28.5	3.981 (2.656, 5.966)***	3.049 (1.999, 4.649)***
Very high/high	41.1	6.981 (4.420, 11.026)***	6.302 (3.899, 10.187)***
Perceived prevalence of HPV infection among female in Hong Kong			
Low/very low/moderate	32.3	1	1
Very high/high	34.1	2.778 (2.056, 3.754)***	2.651 (1.922, 3.657)***
**Perceived benefits of HPV vaccines for preventing and treating diseases related to HPV**			
Perceived efficacy in preventing genital warts			
Not very effective/effective/don’t know	7.7	1	1
Very effective/effective	42.7	8.918 (6.202, 12.824)***	6.750 (4,609, 9.887)***
Perceived efficacy in preventing HPV-induced cancers (penile and anal cancers) (penile			
Not very effective/effective/don’t know	8.1	1	1
Very effective/effective	43.8	8.786 (6.165, 12.522)***	6.817 (4.675, 9.939)***
Perceived efficacy in treating genital warts			
Not very effective/effective/don’t know	20.6	1	1
Very effective/effective	31.8	1.798 (1.303, 2.483)**	1.559 (1.109, 2.191)*
Perceived efficacy in preventing HPV-induced cancers (penile and anal cancers)			
Not very effective/effective/don’t know	19.1	1	1
Very effective/effective	38.8	2.681 (1.933, 3.719)***	2.228 (1.576, 3.151)***
**Perceived barriers to take up of HPV vaccines**			
HPV vaccination is expensive			
Totally disagree/disagree/don’t know	34.6	1	1
Totally agree/agree	31.2	0.315 (0.225, 0.441)***	0.375 (0.262, 0.537)***
HPV vaccines could have sides effects			
Totally disagree/disagree	42.3	1	1
Totally agree/agree	21.0	0.363 (0.254, 0.518)***	0.374 (0.255, 0.548)***
Don’t know	7.7	0.114 (0.074, 0.177)***	0.155 (0.099, 0.244)***
It is embarrassing to take up HPV vaccines			
Totally disagree/disagree	33.0	1	1
Totally agree/agree	19.9	0.506 (0.367, 0.698)***	0.482 (0.342, 0.680)***
Don’t know	3.0	0.063 (0.025, 0.157)***	0.088 (0.035, 0.220)***
It is troublesome to take up HPV vaccines			
Totally disagree/disagree	37.2	1	1
Totally agree/agree	15.0	0.297 (0.210, 0.422)***	0.292 (0.201, 0.424)***
Don’t know	10.1	0.190 (0.117, 0.307)***	0.188 (0.108, 0.329)***
**Perceived self-efficacy to take up of HPV vaccines**			
I am confident that I could take up HPV vaccines if I want to			
Disagree/don’t know	10.3	1	1
Agree	27.3	3.281 (2.090, 5.151)***	2.688 (1.684, 4.288)***
**Cue to action**			
I have watched media reports promoting HPV vaccines among men			
No/unknown	15.0	1	1
Yes	38.7	3.582 (2.644, 4.854)***	2.526 (1.815, 3.516)***
Peer recommended me to take up HPV vaccines			
No/unknown	19.0	1	1
Yes	66.3	8.406 (5.291, 13.354)***	7.108 (4.322, 11.688)***

*p<0.05;

**p<0.01.

***p<0.001

Age group that was not significant in the univariate analysis was not tabulated. ORu: univariate odds ratios. AOR: adjusted OR, odds ratios adjusted for all multivariately significant background variables, including previous exposure to HPV-related services, STD-related symptoms, STD diagnosed in the past year and number of sexual partners.

There were three variables relating to the perceived severity of HPV that significantly influenced subjects’ likelihood of seeking vaccination. Subjects were more likely to get vaccinated if they thought 1) they had a moderate to very high chance of contracting HPV (“moderate chance”: AOR = 4.949, 95% CI = 3.455–7.089; “high/very chance”: AOR = 6.492, 95% CI = 3.807–11.069; reference group was ‘‘low/very low chance”); 2) that HPV could damage their physical health (a moderate chance: AOR = 3.635, 95% CI = 1.787–7.395; a high or very high chance: AOR = 4.607, 95% CI = 2.329–9.114; the reference group, a ‘‘low/very low chance”), and 3) that the chances of their passing on HPV were high or very high (AOR = 2.729, 95% CI = 1.979–3.764; reference group, “a moderate/low/very low chance”). Subjects were further more likely to seek immunization when their knowledge of HPV was at least moderate (“moderate”: AOR = 3.531, 95% CI = 2.357–5.290; “high /very high”: AOR = 13.316, 95% CI = 8.049–22.029; reference group, “low /very low”); when they believed that there was a moderate to very high prevalence of HPV infection among men in Hong Kong (“moderate”: AOR = 3.049, 95% CI = 1.999–4.649; “high /very high: AOR = 6.302, 95% CI = 3.899–10.187; reference group, “low / very low prevalence”), and when they believed that the prevalence of HPV infection among women in Hong Kong was moderate to very high (“moderate”: AOR = 2.651, 95% CI = 1.922–3.657; reference group, “low /very low prevalence”) (Table 4).

Subjects were also more statistically likely to intend to take up vaccination if they thought they could prevent and treat genital warts (AOR = 6.750, 95% CI = 4.609–9.887; AOR = 6.817, 95% CI = 4.675–9.939), as well as penile and anal cancers (AOR = 1.559, 95% CI = 1.109–2.191; AOR = 2.228, 95% CI = 1.576–3.151). Only one item relating to perceived self-efficacy was of statistical significance: responses to “I am confident that I could take up HPV vaccines if I wanted to” (AOR = 2.688, 95% CI = 1.684–4.288) (Table 4).

Several factors reflecting perceived barriers to vaccination were significant: concerns over cost (AOR = 0.375, 95% CI = 0.262–0.537), ‘‘side effects” (AOR = 0.374, 95% CI = 0.255–0.548), “hassle” (AOR = 0.292, 95% CI = 0.201–0.424) and “embarrassment” (AOR = 0.482, 95% 95% CI = 0.342–0.680). Two variables relating to cues to action were significant: media exposure (AOR = 2.526, 95% CI = 1.815–3.516) and peer discussion (or friends: AOR = 7.108, 95% CI = 4.322–11.688). Furthermore, adjusted analysis determined five cognitive composite indicator variables as statistically significant. These related to knowledge of HPV vaccines (AOR = 1.920 to 8.667, p <0.05), perceived susceptibility to infection (AOR = 1.973 to 6.936, p <0.001), perceived benefits from getting immunized (AOR = 6.159 to 9.239, p <0.001), subjects’ sense of the barriers to immunization (AOR = 0.255, p <0.001), and motivations for getting vaccinated (AOR = 3.698 to 3.719, p <0.001) (Table 5).

**Table 5:** Associations between composite cognitive indicator variables) and intention to take up HPV vaccines (n = 1004).

	Row %	ORU (95%CI)	AOR (95%CI)
Knowledge on HPV vaccines (number of items with correct answer)			
0	6.4	1	1
1	14.1	2.460 (1.369, 4.226)**	1.920 (1.074, 3.430)*
≥2	45.3	12.343 (7.820, 18.993)***	8.667 (5.452, 13.779)***
Perceived susceptibility of HPV (number of items with affirmative responses)			
0	13.5	1	1
1	22.3	1.831 (1.273, 2.633)**	1.973 (1.344, 2.896)***
≥2	54.1	7.528 (5.069, 11.179)***	6.936 (4.517, 10.650)***
Perceived benefits to take up HPV (number of items with affirmative responses)			
0	6.3	1	1
1	6.7	1.059 (0.397, 2.282)	0.870 (0.320, 2.367)
2	34.2	7.695 (4.518, 13.109)***	6.159 (3.524, 10.767)***
≥3	45.4	12.343 (8.022, 18.992)***	9.239 (5.851, 14.588)***
Perceived barriers to take up HPV (number of items with affirmative responses)			
0	6.6	1	1
≥1	27.4	0.189 (0.105, 0.338)***	0.255 (0.140, 0.464)***
Cue to action to take up HPV (number of items with affirmative responses)			
0	11.7	1	1
1	40.5	5.134 (3.418, 7.711)***	3.719 (2.420, 5.714)***
≥2	41.2	5.285 (3.706, 7.537)***	3.698 (2.532, 5.400)***

*p<0.05;

**p<0.01.;

***p<0.001

Age group that was not significant in the univariate analysis was not tabulated. ORu: univariate odds ratios. AOR: adjusted OR, odds ratios adjusted for all multivariate significant background variables, including previous exposure to HPV-related services, STD-related symptoms, STD diagnosed in the past year and number of sexual partners. 95% CI: 95% confidence interval.

In addition, the study framed a multiple logistic regression model containing all cognitive composite indicator variables, adjusted for the same significant background variables. It obtained similar results (Table 6).

**Table 6:** Multiple logistic regression—Associations between composite cognitive indicator variables and intention to take up HPV vaccines (n = 1004).

	β	SE	AOR (95%CI)
Knowledge on HPV (number of items with appropriate answer)			
0 or 1	Reference		1
2	0.669	0.603	1.952 (0.599, 6.358)
3	1.271	0.565	3.563 (1.178, 10.778)*
4	0.601	0.571	1.824 (0.596, 5.585)
≥5	0.779	0.529	2.180 (0.773, 6.145)
Knowledge on HPV vaccines (number of items with appropriate answer)			
0	Reference		1
1	0.239	0.341	1.269 (0.651, 2.474)
≥2	1.082	0.302	2.949 (1.632, 5.331)***
Perceived severity of HPV (number of items with affirmative responses)			
0	Reference		1
≥1	0.069	0.223	1.071 (0.692, 1.657)
Perceived susceptibility of HPV (number of items with affirmative responses)			
0	Reference		1
1	0.660	0.232	1.935 (1.227, 3.051)*
≥2	1.531	0.273	4.623 (2.710, 7.886)***
Perceived self-efficacy of HPV (number of items with affirmative responses)			
0	Reference		1
≥1	0.744	0.385	2.104 (0.989, 4.474)
Perceived benefits to take up HPV (number of items with affirmative responses)			
0	Reference		1
1	-0.370	0.535	0.691 (0.242, 1.972)
2	1.423	0.328	4.149 (2.183, 7.885)***
≥3	1.600	0.279	4.954 (2.869, 8.553)***
Perceived barriers to take up HPV (number of items with affirmative responses)			
0	Reference		1
≥1	-1.115	0.369	0.328 (0.159, 0.677)**
Cue to action to take up HPV (number of items with affirmative responses)			
0	Reference		1
1	0.891	0.266	2.438 (1.446, 4.109)**
≥2	0.907	0.228	2.477 (1.584, 3.872)***

*p<0.05;

**p<0.01.

***p<0.001

AOR: adjusted OR, odds ratios after adjusting simultaneously for all involved variables and the significant background variables which include previous exposure to HPV-related services, STD-related symptoms, STD diagnosed in the past year and number of sexual partners. 95% CI: 95% confidence interval. SE: Standard error.

In this model, subjects’ variable degree of knowledge of HPV, understanding of its severity and perceived self-efficacy in getting vaccinated were not statistically significant with regard to whether they intended to get the vaccine or not.

## Discussion

The Centre for Health Protection at the Department of Health in Hong Kong promotes HPV vaccination over an electronic platform. It has secured extensive coverage in the media and has passed to private clinics all the information that is necessary to get vaccinated. Unexpectedly, one third of the study participants (33.8%) had never heard of HPV. About half (50.6%) asserted that HPV was recently discovered.

A similar study suggests men know less about HPV than women [23]. Five HBM-related composite indicator variables (knowledge on HPV vaccines, perceived susceptibility of HPV, perceived benefits to take up HPV vaccines, perceived barriers to take up HPV vaccines and cues to action to take up HPV vaccines) emerged as significant variables in the multiple logistic regression. Broadly, men were not motivated to get vaccinated when they knew nothing about HPV, including their own susceptibility.

### Knowledge of HPV vaccines

The knowledge deficit as regards HPV is likely to prevent men from acting in such a way as minimizes the risks of their either becoming infected or passing on infection. Better knowledge of HPV-vaccines [30] would equip men to make better decisions in the light both of their vulnerability and infectivity [26, 31, 32]. It is important to educate men about HPV, and about their susceptibility to HPV infection in general, so that they can make an informed vaccination decision [30].

### Perceived susceptibility

Just under a third of study (n = 330, 32.9%) participants reported one sexual partner or more in the past year. Of these, 7.5% (n = 75) had STD-related symptoms or an STD diagnosis. Supposing self-reports of sexual activity are true, this indicates that approximately every 1 in 4 participants who had sex was exposed to sexually transmitted infections. More importantly, 3.3% of our participants had multiple sexual partners. Having >2 sexual partners is associated with a greater risk of HPV infection [26, 32]. It would amount to a significant health risk if these men, who knew little about STDs or HPV, engaged in unprotected sex. One possible tactic for raising public awareness would be to make public the number of new HPV cases per annum, and the crude death ratios from the disease, through media releases. While 35.6% of participants perceived that the infectiousness of HPV was “high” or “very high”, only 8% thought they had a high or very high chance of getting HPV—a disconnect found frequently in the literature [33].

### Perceived barriers

In this study, cost (a supposedly expensive price), cognitive and psychological barriers (perceived side effects and embarrassment), and logistical barrier (it being too much trouble) were of statistical significance in whether participants thought there were barriers in the way of getting immunized. It is important to remove these perceived barriers. Currently, the Centre for Health Protection in Hong Kong does not provide HPV vaccination on demand [15]. Hong Kong citizens have to visit their general practitioners and pay for the HPV vaccine at their own cost. It should be worth Hong Kong launching an information program on HPV as the city represents a favorable socio-cultural context in which to inform people about the true extent of the risk [29]. Stakeholders should consider sponsoring HPV vaccination for those at-risk groups in order to improve preventive behavioral outcomes, reduce the financial cost of vaccination to laypersons and to reduce, in turn, the global disease burden [15]. Many men worry about the vaccine’s side effects [34]. Primary care physicians are always the first point of contact with HPV-infected individuals, and these physicians in private clinics should educate those in need and at risk about how they can get immunized [23]. The easy accessibility of the vaccine will potentially lower logistical barriers and make more young men prepared to get the HPV vaccine [35].

Suffering from STD-related symptoms or being diagnosed with an STD is embarrassing. In order to reduce the social stigma attached to HPV vaccines, the Department of Health, Hong Kong, could run a positive campaign on their benefits in collaboration with university health-services [35], This provides a good opportunity to educate students about the prevention of HPV infections and promotion of HPV vaccines. Good doctor-student relationships in the university may also mitigate embarrassment, increase students’ understanding of the transient side-effects of taking up the HPV vaccine and increase the likelihood they will choose to get vaccinated [36]. Prophylactic vaccines, whose impact is greatest when administered prior to disease exposure [37], and thus HPV vaccination targeted at young adults should be integrated into university health service as part of a vaccination package. Young adults are a challenging population to reach with preventive health services [37, 38], but the university is a promising environment in which to offer integrated provision and to seek to bridge young people’s knowledge gaps in these areas. HPV vaccination on-campus may facilitate vaccine uptake and boost baccalaureate students’ willingness to take the jabs [23].

### Cues to action

How subjects responded to cues is one of the key determinants of their health-related behaviors. Our study suggested that, in disposing men to get vaccinated for HPV, two out of three types of cues to action (media and peer information) were significant. Although medical doctors are actively involved in primary, secondary and tertiary health promotion, doctors’ recommendations were not a significant factor in the multiple logistic regression model. This may be because half of the participants (50.2%) had never been exposed to HPV-related services and would in all probability not have known what doctors had to say about HPV vaccination. Nevertheless, health professionals play a pivotal role in health promotion [39]. Peer education and peer testimonials among young adults also count as a significant variable in health promotion campaigns [29]. Due to advances in technology, young adults are more likely to turn to electronic media in looking for health information [36, 40]. Promotional campaigns should then adopt a user-friendly electronic platform to deliver their message to young adults. A social media campaign, for instance, may consider recruiting evidently healthy celebrities to promote vaccination [41].

### Perceived self-efficacy and perceived benefits

Perceived self-efficacy was non-significant, possibly due to the small variation in item responses [29], in that about 77% of the participants were confident they could take up HPV vaccines if they wanted to. 82% claimed full control on whether they took up HPV vaccines (Table 3). We found the high percentage of perceived self-efficacy encouraging because self-efficacy is a strong predictor of vaccination intentions and uptake [42]. When an individual has high self-efficacy on matters of health behaviors, they may be more easily influenced by health information [39]. We speculate that providing a free or discounted HPV vaccines programme through the University Health Service, stakeholders could cue baccalaureate students to take preventive measures and reduce their risky health behaviors. If the students were better informed of the benefits of getting vaccinated against HPV (e.g. preventing HPV infections and reducing the likelihood of genital warts and anal, penile and cervical cancers), they would be less embarrassed to take the course of injections, and may be prepared to go some way towards its total cost. Perceived benefits may outweigh perceived barriers in taking up HPV vaccination.

In summary, our research hypotheses 1, 2 and 3 could be established. Participants who had higher perceived susceptibility, higher perceived severity and increased level of knowledge on HPV were significantly correlated with their acceptability and intention to take up the HPV vaccination. However, we would reject our hypothesis 4 that perceived self-efficacy was positively associated with the intention to take up the HPV vaccination.

This study has some limitations. First, the samples recruited in this study may be subject to selection bias as convenience samples without a sampling frame were used. Second, cross-sectional data cannot establish the causal relationship between risky sexual behavior and willingness to get vaccinated for HPV. Third, participants were asked about their behavioral intention to take up the HPV vaccine, rather than their actual behavior being scrutinized. Fourth, no validated scale can be used to accurately measure people’s willingness to get vaccinated for HPV. As a result, questions were constructed with reference to a similar study conducted in Hong Kong [29]. Lastly, issues of social desirability may over-estimate the overall likelihood of people getting vaccinated against HPV [23]. Notwithstanding these limitations, the findings emerging from this study may provide useful information to guide future research into HPV vaccination and prevention.

## Conclusion

HPV is a significant public health concern for university-aged men. This highly infectious disease can lead to serious health outcomes. This study has uncovered the prevalence of male university students’ willingness to be vaccinated against HPV in Hong Kong, unveiling significant composite factors associated with the perceived acceptability of HPV vaccines. The study cohort seriously underestimate the chance they will get HPV as evidenced by our findings emerging that less than one quarter of our participants had the intention to take up HPV vaccine. Such a disconnect follows from a knowledge deficit. Participants’ sense of their susceptibility mean that the disadvantages (or inconvenience) of taking the course of prophylactics outweigh any benefits they see themselves incurring. Too many barriers stand in the way of their getting immunized; and there are too few cues to action motivating them to self-efficacious preventative behavior. Healthcare providers should focus on removing these psychological and financial barriers before progressing to the next stage of any health promotion campaign. Health policy-makers should consider a sponsored trial of a free HPV vaccination programme of an at-risk population to examine the cost-effectiveness of this preventative measure.

### Ethics approval

The study was approved by the Human Subjects Ethics Sub-Committee, and the Institutional Review Board of the Hong Kong Polytechnic University (Reference No: HSEARS20150513004). Our Human Subjects Ethics Sub-Committee approved the lack of parental consent for participants who were under 18 to participate in this study.

## Supporting information

S1 File This is the HPV dataset File. (XLSX)

S2 File This is the HPV instrument File. (DOCX)
