# Supplementary material for: Acceptability of HPV vaccines and associations with perceptions related to HPV and HPV vaccines among male baccalaureate students in Hong Kong
Source: PLoS One. 2018 Jun 18;13(6):e0198615. doi: 10.1371/journal.pone.0198615 (PMC6005511; doi:10.1371/journal.pone.0198615)
Supplement: S2 File — (DOCX) [file pone.0198615.s002.docx]

**Acceptability of HPV Vaccines and Associations with Perceptions Related to HPV and HPV Vaccines Among Male Baccalaureate Students in Hong Kong**

Part I：Sociodemographic Data

1. How old are you？

□_1_ Below 16 years old □_2_ 17-26 years old □_3_ Above 27 years old -

1. What subject do you study?

□_1_Health-related subject □_2_ Non-Health-related subject

1. Have you ever exposed to any HPV service-related information, such as poster, talk or pamphlet?

□_1_Yes  □_2_ No

1. In the past year, have you suffered from the following symptoms of sexual transmitted disease?

Pain in urination □Yes □No

Abnormal urethra excretion □Yes □No

Skin breakdown in sexual organ or annul □Yes □No

Abnormal growth in sexual organ or annul □Yes □No

1. In the past year, have you been diagnosed with sexual transmitted disease?

□_1_ Yes □_2_ No

1. In the past six months, how many sex partners did you have?

□_1_ 0  □_2_ 1  □_3_ More than 1

Part II：HPV-related Knowledge

**HPV is the short term of Human Papillomavirus. Some specific types of HPV are associated with the cause of reproductive tract malignancy, such as cervical cancer.**

1. Have you ever heard of HPV or Human Papillomavirus before doing this questionnaire?

□_1_Yes □_2_No

1. Whether male or female could be infected by HPV?

□_1_Only male

□_2_Only female

□_3_Both male and female

□_4_ I Don't know

1. Which of the followings are the route of infection of HPV? (Tick those boxes that apply)

□_1_Sex □_2_Droplet □_3_ Water-borne □_4_Food

□_5_Mother-to-infant □_6_Mosquito-borne □_7_Rodent-borne □_8_Poultry-borne

□_9_Fecal □_10_ Contact □_11_Others:________

□_12_I Don't know

1. Do you agree with the following statements?

|  | **Agree** | **Disagree** | **I Don't know** |
| --- | --- | --- | --- |
| HPV was newly found in the past few years | □_1_ | □_2_ | □_3_ |
| HPV could be controlled by antibiotics | □_1_ | □_2_ | □_3_ |
| HPV is unlikely to be totally cured | □_1_ | □_2_ | □_3_ |
| HPV is hereditary | □_1_ | □_2_ | □_3_ |
| HPV has high mortality rate, >5% | □_1_ | □_2_ | □_3_ |
| HPV infection can cause genital warts | □_1_ | □_2_ | □_3_ |
| HPV infection can cause anal cancers | □_1_ | □_2_ | □_3_ |
| HPV infection can cause penile cancers | □_1_ | □_2_ | □_3_ |

How do you perceive the following statements?

|  | **Very High** | **High** | **Moderate** | **Low** | **Very Low** |
| --- | --- | --- | --- | --- | --- |
| How do you perceive your chance of contacting HPV in the future？ | □_1_ | □_2_ | □_3_ | □_4_ | □_5_ |
| How do you perceive the damages of HPV infection to physical health？ | □_1_ | □_2_ | □_3_ | □_4_ | □_5_ |
| How do you perceive the infectivity of HPV？ | □_1_ | □_2_ | □_3_ | □_4_ | □_5_ |
|  | **Very High** | **High** | **Moderate** | **Low** | **Very Low** |
| How do you perceive your knowledge on HPV？ | □_1_ | □_2_ | □_3_ | □_4_ | □_5_ |
| How do you perceive the chance of male in rate of HPV infection？ | □_1_ | □_2_ | □_3_ | □_4_ | □_5_ |
| How do you perceive the chance of female rate of HPV infection？ | □_1_ | □_2_ | □_3_ | □_4_ | □_5_ |

Part III：HPV vaccine-related knowledge

1. Is there any HPV vaccine in the market to reduce male in HPV infection chance？

□_1_Yes □_2_No（Jump to Q.14） □_3_ I Don't know

1. How much does each HPV vaccine cost in the market?

□_1_Below 800 HKD □_2_ 800-1500 HKD □_3_Above 1500 HKD □_4_ I Don't know

13. What is the effective number of dose in vaccination?

□_1_ One □_2_ Two □_3_ Three □_4_ I Don't know

1. What is the protection period of vaccination?

□_1_Around 1 year □_2_ 2-5 years 　□_3_ 5-10 years　　 □_4_ Above 10 years

□_5_ Entire life　　 □_6_ I Don't know

1. What is the suggested age group of HPV vaccination in Hong Kong ？

□_1_ Below 9 years old □_2_ 9-26 years old □_3_ Above 27 years old □_4_All age group

□-_5_ I Don't know

Part IV：View towards HPV vaccine efficacy

1. The followings are your view towards vaccine efficacy：

|  | **Very Effective** | **Effective** | **Not Very Effective** | **Not Effective** | **I Don't Know** |
| --- | --- | --- | --- | --- | --- |
| HPV vaccine efficacy in preventing **genital warts** | □_1_ | □_2_ | □_3_ | □_4_ | □_5_ |
| HPV vaccine efficacy in preventing **HPV induced cancers** | □_1_ | □_2_ | □_3_ | □_4_ | □_5_ |
| HPV vaccine efficacy in preventing **STD other than genital warts** | □_1_ | □_2_ | □_3_ | □_4_ | □_5_ |
| HPV vaccine efficacy in treating **genital warts** | □_1_ | □_2_ | □_3_ | □_4_ | □_5_ |
| HPV vaccine efficacy in treating **HPV induced cancers** | □_1_ | □_2_ | □_3_ | □_4_ | □_5_ |

| 1. The followings are your views towards HPV vaccination： |  |  | |  |  | |  |
| --- | --- | --- | --- | --- | --- | --- | --- |
|  | **Totally Disagree** | **Disagree** | | **Agree** | **Totally Agree** | | **I Don't Know** |
| HPV vaccination is expensive | □_1_ | □_2_ | | □_3_ | □_4_ | | □_5_ |
| HPV vaccination could have side effects | □_1_ | □_2_ | | □_3_ | □_4_ | | □_5_ |
| It is embarrassing to take up HPV vaccines | □_1_ | □_2_ | | □_3_ | □_4_ | | □_5_ |
| It is troublesome to take up HPV vaccines | □_1_ | □_2_ | | □_3_ | □_4_ | | □_5_ |
| Private doctors do not provide HPV vaccination | □_1_ | □_2_ | | □_3_ | □_4_ | | □_5_ |
| Public hospitals | □_1_ | □_2_ | | □_3_ | □_4_ | | □_5_ |
| Taking up HPV vaccine may be seen as a sign of promiscuity | □_1_ | □_2_ | | □_3_ | □_4_ | | □_5_ |
| Male usually won't agree to have HPV vaccination | □_1_ | □_2_ | | □_3_ | □_4_ | | □_5_ |
| I am confident that I could take up HPV vaccines if I want to | □_1_ | □_2_ | | □_3_ | □_4_ | | □_5_ |
| I have full control on whether taking up HPV vaccine | □_1_ | □_2_ | | □_3_ | □_4_ | | □_5_ |
|  | **Yes** | | **No** | | | **I Don't Know** | |
| 1. I have watched media reports promoting HPV vaccines | □_1_ | | □_2_ | | | □_3_ | |
| 19. Doctor recommended me to take up HPV vaccines | □_1_ | | □_2_ | | | □_3_ | |
| 20. Peer recommended me to take up HPV vaccines | □_1_ | | □_2_ | | | □_3_ | |
|  |  | |  | | |  | |

Part V：Attitude

1. According to your understanding, what is your chance in taking up HPV vaccination?

□_1_ Must

□_2_ High Probability

□_3_ Low Probability

□_4_ Must Not

□_-5_ I Don't Know

The survey is completed. Thank you for your participation.
